# Pleural small cell carcinoma with massive pleural effusion: A case report

**DOI:** 10.1097/MD.0000000000018251

**Published:** 2019-11-27

**Authors:** Jong Geol Jang, Min Hye Jang, June Hong Ahn

**Affiliations:** a Department of Pulmonology and Allergy, Department of Internal Medicine; b Department of Pathology, Yeungnam University Medical Center, Daegu, Republic of Korea.

**Keywords:** extrapulmonary, pleura, pleural effusion, small cell carcinoma

## Abstract

**Rationale:**

Small cell carcinoma (SCC) occurs mostly in the lung, and small cell lung cancer accounts for 13% of newly diagnosed lung cancers. Only 2.5% of SCC occurs in extrapulmonary sites, and SCC of pleural origin is especially very uncommon.

**Patient concerns:**

An 85-year-old man presenting with progressive dyspnea for more than 7 days.

**Diagnoses:**

Computed tomography scan of the chest showed massive pleural effusion and diffuse nodular thickening of the pleura on the right chest. Sonography-guided needle biopsy of the pleural mass was performed and histologic and immunohistochemical findings revealed SCC. Since no parenchymal lung lesion was observed, the patient was finally diagnosed with SCC of the pleura (SCCP).

**Interventions:**

Due to the patient's old age and poor performance status, chemotherapy was not performed and only drainage of pleural effusion was conducted for symptom relief.

**Outcomes:**

Dyspnea improved after pleural effusion drainage. The patient was discharged and transferred to a local medical center for hospice care.

**Lessons:**

Although primary SCCP is extremely rare, SCCP should also be considered as well as mesothelioma in case of presence of a pleural-based mass with massive pleural effusion.

## 1 Introduction

Small cell carcinoma (SCC) occurs mostly in the lung and small cell lung cancer accounts for 13% of newly diagnosed lung cancers.^[1]^ Only 2.5% of SCC occurs in extrapulmonary sites.^[2]^ Extrapulmonary SCC (EPSCC) was first reported in 1930 and there have been many reports of cases since then.^[3]^ Since EPSCC is rare, its natural clinical course and optimal therapy has not been determined and it is still underdiagnosed and confused with metastatic small cell lung cancer (SCLC).^[4]^ The SCC of the pleura (SCCP) is especially rare and only a few cases have been reported so far.^[5–9]^ Here, we report a recent case of SCCP.

## 2 Case report

An 85-year-old man with a 60 pack-year history of cigarette smoking was referred to our hospital due to progressive dyspnea for 7 days. He was a farmer with an unknown history of asbestos exposure. He had a medical history of type 2 diabetes mellitus and old treated tuberculosis. On physical examination, decreased breath sound in the right lung was observed. His blood cell counts and biochemistries were as follows; hemoglobin 13.8 g/dL, white cell count 6280/μL, platelet count 404,000/μL, total protein 4.18 g/dL, albumin 2.66 g/dL. Serological tumor markers were within normal limits (carcinoembryonic antigen [CEA] = 2.46 ng/mL, CYFRA 21-1 = 2.88 ng/mL). However, the level of CEA in pleural effusion was found to be elevated to 12.75 ng/mL (reference range 0–10 ng/mL).

**Figure 1:**
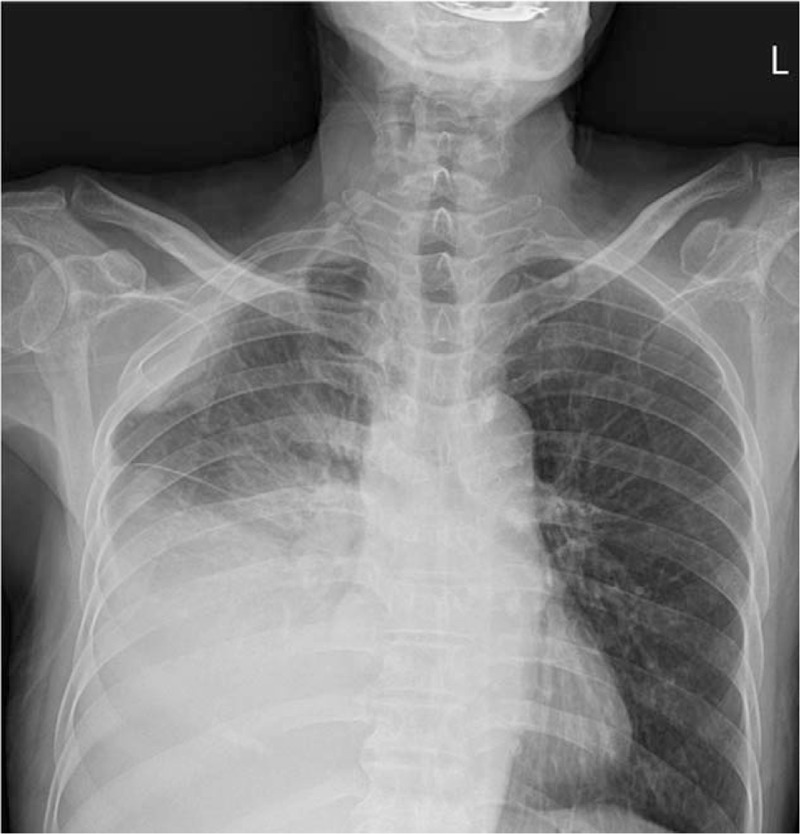
Chest X-ray revealed pleural effusion at the right side with pleural based nodular opacity.

**Figure 2:**
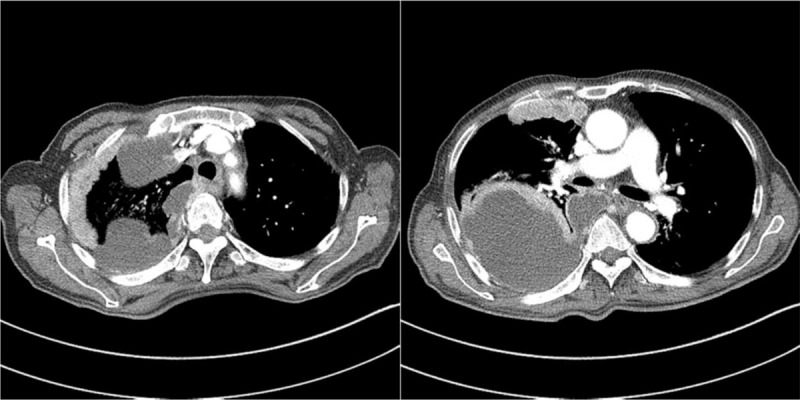
Chest computed tomography revealed massive right pleural effusion and diffuse nodular thickening with passive atelectasis in the right middle lung and right lower lung fields.

The chest X-ray image revealed pleural effusion at the right side with pleural-based nodular opacity (Fig. 1). Computed tomography (CT) scan of the chest showed massive right pleural effusion and diffuse nodular thickening with passive atelectasis in the right middle lung and right lower lung fields. However, no mass-like lesion was observed in the lung parenchyme (Fig. 2).

**Figure 3:**
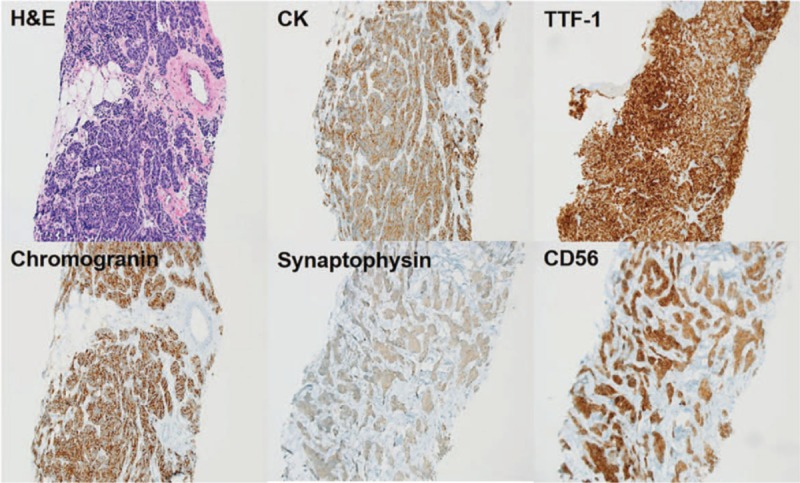
Microscopic photo and immunohistochemistry of pleural small cell carcinoma. Microscopically, tumor showed small nests or trabecular architectural pattern. The tumor cells had oval to spindle nuclei with scant cytoplasm. The nuclear chromatin is fine granular with inconspicuous nucleoli which is the typical nuclear feature of neuroendocrine tumor. Mitosis was frequently observed. The tumor cells showed positive expression of chromogranin, synaptophysin, and CD56. And it also showed positive expression of CK with dot-like paranuclear pattern. Interestingly tumor cells showed nuclear TTF-1 expression. (original magnification ×200).

Histological sample obtained by sonography-guided needle biopsy of pleural mass revealed malignant cells, suggestive of SCC (Fig. 3). Final diagnosis was confirmed by immunohistochemical (IHC) analysis which revealed that the tumor cells were positive for neuroendocrine markers, such as CD56, chromogranin A, synaptophysin, Cytokeratin (CK), and thyroid transcription factor (TTF-1) but negative for D2–40, CK20, CDX2, and HBME1 (Fig. 3). Subsequently, a diagnosis of pleural small cell carcinoma was made. Brain Magnetic Resonance Imaging and fused 18-fluorodeoxyglucose positron emission tomography-computed tomography (18-FDG-PET/CT) did not show any mass or lymphadenopathy. Chemotherapy was not performed due to the patient's old age and poor performance status. Only drainage of pleural effusion was performed for relieving dyspnea.

As this study is a clinical case report, no ethical committee approval was required, which is in compliance with the institutional and national policies concerning research approvals. The family of patient was informed that clinical details and images concerning the case would be submitted for publication, and they provided consent.

## 3 Discussion

SCC usually occurs in the lung, but it can also occur in various other organs.^[2]^ EPSCC was first reported in 1930 and is now considered as a clinicopathologic entity distinct from small cell lung cancer.^[3]^ Previous studies reported that EPSCC originated from various organs, and the common primary sites were the gastrointestinal tract, cervix, urinary bladder, head, and the neck in the cases reported.^[4,10–12]^ However, SCCP is extremely rare, and only 5 cases have been reported so far, according to our knowledge.^[5–9]^

Mesothelioma is an important differential diagnosis in which pleural tumor without parenchymal lung lesion is found.^[8]^ Mesothelioma frequently shows pleural effusion in CT scanning at presentation,^[13]^ but in SCLC, pleural effusion is an infrequent clinical finding.^[13]^ One study reported that only 3% (8/256) of patients with SCLC had clinically significant pleural effusion.^[14]^ Our case was initially considered as mesothelioma because the CT scan showed massive pleural effusion and pleural-based mass without parenchymal lung lesion. However, it was distinguished from mesothelioma by microscopic features and the expression in IHC staining which was positive for chromogranin, synaptophysin, and CD56 (which is in favor of SCC) but negative for anti-mesothelial cell antibody.^[15]^ Though the expression of TTF-1 in our case was positive, it is controversial to some degree. Noguchi et al mentioned that TTF-1 is not presented in EPSCC,^[16]^ while another study reported that it is presented in 42% of EPSCC.^[17]^ To diagnose EPSCC, evidence of intrapulmonary lesion which can be the primary tumor^[5]^ should not be found. In the present case, there was no evidence of any intrapulmonary malignancy in Chest CT scan and 18-FDG-PET/CT.

The clinical course of EPSCC is aggressive and often recurs after treatment.^[10,18]^ The prognosis of EPSSC depends on the extent of the disease and the organ of origin.^[18]^ In 2 previous case series, the median survival times of patients with extensive disease (ED) were 7 months and 12 months, respectively.^[11,12]^ Multimodal treatment, including surgery or radiotherapy followed by chemotherapy, is the recommended treatment in patients with limited disease (LD),^[4,11,12,19]^ while in patients with ED, platinum-based chemotherapy is usually performed.^[19]^ Although 2 case series had reported partial and complete responses to platinum-based chemotherapy, the prognosis and response rate of SCC of the pleura is unknown due to its extremely low prevalence. We also considered cisplatin-based chemotherapy, which could not be performed because of the poor performance status of the patient.

An interesting finding in our case is the presence of massive pleural effusion, unlike the previous cases. In case of pleural-based mass with massive pleural effusion, SCC of the pleura and mesothelioma should be considered.

In conclusion, though primary SCCP is extremely rare, SCCP should also be considered as well as mesothelioma in case of presence of a pleural-based mass with massive pleural effusion. As there are no randomized trials for treating SCCP, an individualized treatment plan is important, and observational strategy might be an appropriate option in patients with poor performance status.

## Author contributions

**Conceptualization:** Jong Geol Jang, June Hong Ahn.

**Resources:** Min Hye Jang, June Hong Ahn.

**Writing – original draft:** Jong Geol Jang.

**Writing – review & editing:** June Hong Ahn.
